# Prediction of nocturnal hypoglycemia unawareness by fasting glucose levels or post-breakfast glucose fluctuations in patients with type 1 diabetes receiving insulin degludec: A pilot study

**DOI:** 10.1371/journal.pone.0177283

**Published:** 2017-07-06

**Authors:** Hiroshi Takahashi, Rimei Nishimura, Yoshiko Onda, Kiyotaka Ando, Daisuke Tsujino, Kazunori Utsunomiya

**Affiliations:** Division of Diabetes, Metabolism and Endocrinology, Department of Internal Medicine, Jikei University School of Medicine, Tokyo, Japan; Weill Cornell Medical College Qatar, QATAR

## Abstract

**Objective:**

To evaluate whether nocturnal asymptomatic hypoglycemia (NAH) can be predicted by fasting glucose levels or post-breakfast glucose fluctuations in patients with type 1 diabetes (T1D) receiving insulin degludec.

**Methods:**

Patients with T1D receiving insulin degludec underwent at-home CGM assessments. Indices for glycemic variability before and after breakfast included fasting glucose levels and the range of post-breakfast glucose elevation. For comparison, the patients were classified into those with NAH and those without. The optimal cut-off values for the relevant parameters were determined to predict NAH using ROC analysis.

**Results:**

The study included a total of 31 patients (mean HbA1c values, 7.8 ± 0.7%), and 16 patients (52%) had NAH. Those with NAH had significantly lower fasting glucose levels than did those without (82 ± 48 mg/dL vs. 144 ± 69 mg/dL; *P* = 0.009). The change from pre- to post-breakfast glucose levels was significantly greater among those with NAH (postprandial 1-h, *P* = 0.028; postprandial 2-h, *P* = 0.028). The cut-off values for prediction of NAH were as follows: fasting glucose level <84 mg/dL (sensitivity 0.80/specificity 0.75/AUC 0.80; *P* = 0.004), 1-h postprandial elevation >69 mg/dL (0.75/0.67/0.73; *P* = 0.033), and 2-h postprandial elevation >99 mg/dL (0.69/0.67/0.71; *P* = 0.044).

**Conclusions:**

The results suggest that fasting glucose level of < 84 mg/dL had approximately 80% probability of predicting the occurrence of NAH in T1D receiving insulin degludec. It was also shown that the occurrence of hypoglycemia led to greater post-breakfast glucose fluctuations and steeper post-breakfast glucose gradients.

## Introduction

The aims of diabetes treatment are preventing the onset and progression of diabetic complications through glycemic and metabolic control and maintaining quality of life (QOL) and lifespan for affected individuals. The DCCT/EDIC study, a large-scale clinical study on patients with type 1 diabetes, has clearly demonstrated that tight glycemic control through intensive insulin therapy significantly reduces the onset and progression of diabetic complications [1–4].

However, “the lower the HbA1c, the better the prognosis” does not necessarily apply to patients with type 1 diabetes, who are reportedly at a greater (rather than smaller) risk of all-cause and cardiovascular mortality even when their HbA1c values are controlled to 6.9% or lower [5]. This is likely due to the increased risk of hypoglycemia associated with tight glycemic control aimed at lowering HbA1c [6–8]. Indeed, the frequency of hypoglycemia showed a three-fold increase among those assigned to tight glycemic control in the DCCT/EDIC study [2,9].

Hypoglycemia is reported to account for 2% to 4% of all deaths among patients with type 1 diabetes, and can even be as high as 6% to 13% according to some reports [10–12]. Indeed, serious hypoglycemia is thought to be associated with sudden death or the “Dead in Bed” syndrome [13–16], where hypoglycemia is assumed to adversely affect the cardiovascular system, thus inducing arrhythmia or QT prolongation [17]. More importantly, the onset of nocturnal hypoglycemia is reported to lead to sympathetic nerve activation, followed by excessive compensatory vagal activation, resulting in bradycardia and life-threatening arrhythmia [18]. Again, frequent hypoglycemic episodes are associated with a significantly increased risk for mortality among patients with type 1 diabetes who have a history of cardiovascular disease [19]. Otherwise, serious hypoglycemia is shown to significantly increase the risk for mortality among patients with type 1 and type 2 diabetes alike, irrespective of their history of cardiovascular disease [20].

Given these considerations, it is clear that attention needs to be focused on improving HbA1c while simultaneously minimizing the occurrence of hypoglycemia in order to help patients maintain their QOL and lifespan. Again, while a number of continuous glucose monitoring (CGM)-based studies have reported that patients with type 1 diabetes are often susceptible to nocturnal hypoglycemia [21–23], asymptomatic episodes of nocturnal hypoglycemia are difficult to capture because they occur without the use of a CGM, which is not readily available for routine use in an outpatient setting.

In an earlier study of inpatients with patients with type 1 diabetes receiving insulin detemir or insulin glargine, we reported that fasting glucose levels that are lower than 135 mg/dL or an acute increase in post-breakfast glucose levels are predictive of nocturnal asymptomatic hypoglycemia in patients with type 1 diabetes [24].

Of the insulin formulations that are available, insulin degludec is an ultra-long-acting basal insulin whose efficacy is shown to be sustained over more than 24 hours, unlike insulin detemir or insulin glargine [25]. A number of studies have shown that insulin degludec provides comparable glycemic control to insulin glargine, but is superior to insulin glargine in reducing the occurrence of nocturnal hypoglycemia [26–28]. However, no studies thus far have investigated the relationship between glycemic variability and nocturnal asymptomatic hypoglycemia in patients receiving insulin degludec by using CGM.

Therefore, we used CGM to investigate whether fasting glucose levels and post-breakfast glucose excursions could predict the occurrence of nocturnal asymptomatic hypoglycemia in outpatients with patients with type 1 diabetes who are receiving basal-bolus insulin therapy and incorporating insulin degludec as long-acting insulin.

## Patients and methods

This study included patients with type 1 diabetes receiving basal-bolus insulin therapy that incorporated insulin degludec as a long-acting soluble insulin at our outpatient clinic. Patients were considered eligible if they were between 20 and 80 years old and if they had HbA1c values between 6.9% and 9.0%. **Patients were recruited from April 2014 to March 2015, and** were excluded if they met any of the following criteria: 1) they had type 2 diabetes; 2) they were receiving oral hypoglycemic agents (OHAs); 3) they had serious ketoacidosis or diabetic coma at the time of recruitment; 4) they had serious infections, had undergone/were undergoing surgery, or had serious traumatic injury; 5) they had hepatic impairment (AST/ALT, >2.5 times upper limit of normal or presence of hepatic cirrhosis); 6) they had renal impairment (creatinine, ≥1.3/1.2 mg/dL in men/women); 7) they had severe cardiovascular or pulmonary disease such as shock, cardiac failure, myocardial infarction, pulmonary embolism, or any other condition or disease associated with hypoxia; 8) they were in a state of malnutrition, starvation, or debility or pituitary malnutrition or had adrenal dysfunction; 9) they were habitual heavy drinkers; 10) they were dehydrated or had gastrointestinal diseases, such as diarrhea or vomiting, associated with the risk of dehydration; 11) they had malignancy; 12) they had, or were likely to have, allergy to insulin or similar drugs; 13) they were, or likely to be, pregnant; and 14) they were judged by the attending physician to be ineligible for the study. The insulin dosing schedule was adjusted to fit the lifestyle of each patient, and the determination of the insulin dose was left to the discretion of the treating physician and the patient. All patients were enrolled in the study after signing of informed consent to study participation.

**Fig 1:**
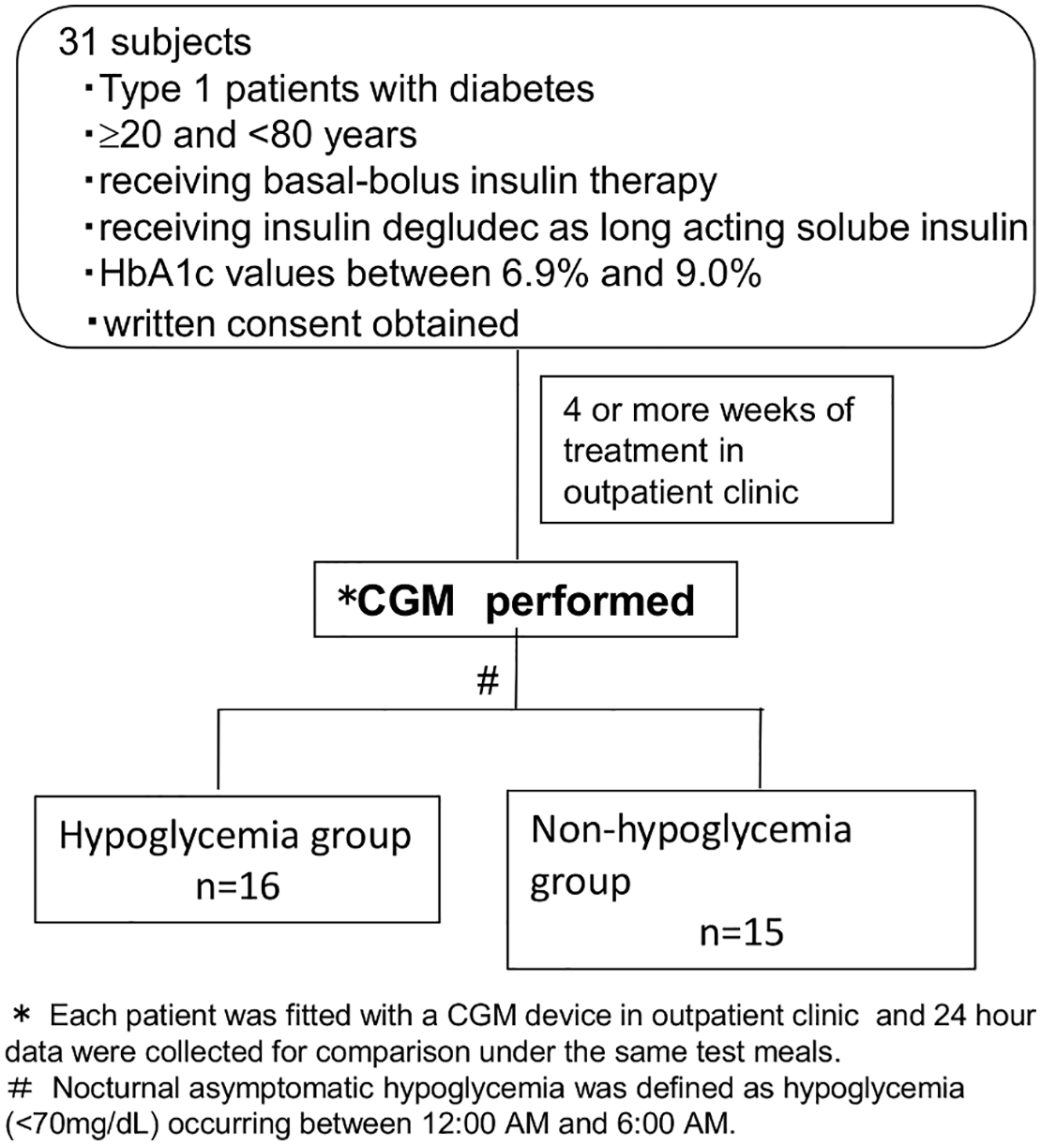
Study design.

Fig 1 shows the study design. The CGM evaluation was conducted using a Medtronic iPro^™^2 CGM System, with Medtronic Enlite^™^ sensors (Medtronic Minimed, Northridge, CA, USA). The CGM was fitted in the outpatient clinic and conducted in the home setting after the patient received insulin degludec for four weeks or more. Analyses were performed on CGM data obtained from all patients during the first night and first full day in which they had been given test meals (breakfast, lunch, and dinner on the day of assessment, as well as dinner on the previous day). The dinner given on day 1 accounted for 591.3 kcal of energy (carbohydrates, 65.1%; proteins, 15.9%; and lipids, 19.0%). Of the total calorie intake on day 2 (1835.3 kcal), breakfast accounted for 616 kcal (carbohydrates, 63.6%; proteins, 16.5%; and lipids, 19.9%).

CGM data obtained from 12:00 AM to 12:00 PM on day 2 were included for analysis, with the exception of those from patients who took glucose on becoming aware of hypoglycemia.

This study was used the same analysis as that in the previous study [24], however, using data from different individuals with type 1 diabetes receiving insulin degludec treatment (insulin detemir or glargine was used in previous study [24]). The occurence of hypoglycemia (<70 mg/dL) between 12:00 AM and 6:00 AM was defined as nocturnal asymptomatic hypoglycemia. All patients were measured for nocturnal glucose nadirs and duration of nocturnal asymptomatic hypoglycemia. Indices for glycemic variability before and after breakfast included the following: I) fasting glucose levels; II) post-breakfast glucose levels (peak, postprandial 1- and 2-hour glucose levels); III) post-breakfast glucose elevation defined as the change from pre- to post-breakfast peak glucose levels (from pre-prandial to peak, 1- and 2-hour postprandial glucose levels); and IV) post-breakfast glucose concentration gradient (from pre-prandial to peak, 1- and 2-hour postprandial levels), all of which were compared between those with hypoglycemia and those without by using the unpaired student's two sampled *t*-test. Additionally, a receiver operating characteristic (ROC) analysis was performed to investigate whether indices I to IV may allow the occurrence of hypoglycemia to be predicted, and if so, to determine the appropriate cut-off values for these indices that allow hypoglycemia to be predicted. Fisher’s exact test (two-tailed test) was performed to investigate the frequency of hypoglycemia depending on the timing of insulin injection. Multivariate logistic regression analysis was also conducted by using the forward selection method (Likelihood Ratio) to identify predictors of nocturnal hypoglycemia unawareness, with duration of diabetes, sex, HbA1c, BMI, total daily dose of insulin, basal insulin ratio, and timing of the insulin injection serving as independent variables.

The IBM SPSS Version 22.0 (IBM Corp. Released 2013. IBM SPSS Statistics for Windows, Version 22.0. Armonk, NY: IBM Corp.) was used to perform all statistical analyses. All the data were expressed as means ± standard deviations (SD). A *P* value of less than 0.05 was considered to be statistically significant (two-tailed).

This study was conducted as a sub-analysis of the Jikei-Evaluation of the basal insulin analogue, Tresiba^®^(insulin degludec), a new basal insulin analogue for (nocturnal) glycemic variability as compared to an existing basal insulin analogue using continuous glucose monitoring in basal-bolus treatment for patients with type 1 diabetes—JET1 Study (UMIN000013817) [29, 30] with the approval of the Institutional Review Board of Jikei University School of Medicine, Tokyo, Japan.

## Results

Thirty-one patients with type 1 diabetes receiving basal-bolus therapy of insulin degludec underwent at-home CGM assessments. None of the patients took glucose upon becoming aware of nocturnal hypoglycemia. The patient background characteristics were: males, 14 (45.2%); age from 24 to 68 years old (mean age, 46.9 ± 11.4 years old [S1 File]); HbA1c, 7.8 ± 0.7%; BMI, 22.4 ± 3.0 kg/m2; and duration of diabetes, 19.5 ± 9.7 years (Table 1). Basal insulin was used once in the evening or before bedtime by 17 patients (54.8%) and in the morning by 14 patients (45.2%).

**Table 1:** Patient profile and parameters for glycemic variability compared between hypoglycemic and non-hypoglycemic patients.

	Overall	Hypoglycemic	Non-hypoglycemic	*P* value*
Patients tested (n)	31	16	15	
Age (years)	46.9 ± 11.4	49.2 ± 12.5	44.5 ± 10.1	*P* = 0.265
HbA1c (%)	7.8 ± 0.7	7.8 ± 0.7	7.8 ± 0.7	*P* = 0.894
Body Mass Index (kg/m^2^)	22.4 ± 3.0	22.5±3.4	22.4±2.5	*P* = 0.930
Duration of diabetes (years)	19.5 ± 9.7	23.9 ± 10.3	14.9 ± 6.6	*P* = 0.008**
Total daily insulin dose (TDD) (U/kg/day)	0.65 ± 0.20	0.65 ± 0.19	0.65 ± 0.22	*P* = 0.935
Total daily basal insulin dose (U/kg/day)	0.25 ± 0.10	0.27 ± 0.11	0.23 ± 0.09	*P* = 0.175
Total daily bolus insulin dose (U/kg/day)	0.40 ± 0.16	0.38 ± 0.15	0.42 ± 0.17	P = 0.467
Before breakfast	0.12 ± 0.05	0.11 ± 0.05	0.13 ± 0.06	P = 0.339
Before lunch	0.13 ± 0.07	0.12 ± 0.06	0.14 ± 0.07	P = 0.608
Before dinner	0.15 ± 0.06	0.14 ± 0.06	0.15 ± 0.07	P = 0.609
Basal insulin ratio (%)	39.1 ± 11.9	42.1 ± 11.6	35.8 ± 11.6	*P* = 0.139
Nighttime glucose nadir levels (mg/dL)	87 ± 53	53 ± 10	124 ± 56	*P*<0.001**
Nighttime duration of hypoglycemia (min)	80 ± 121	155 ± 129	0 ± 0	*P*<0.001**
Fasting glucose levels (mg/dL)	112 ± 66	82 ± 48	144 ± 69	*P* = 0.009**
Post-breakfast glucose levels (mg/dL)				
Peak	204 ± 60	214 ± 62	194 ± 59	*P* = 0.358
Postprandial 1-h	179 ± 55	169 ± 46	190 ± 62	*P* = 0.310
Postprandial 2-h	192 ± 65	195 ± 79	188 ± 49	*P* = 0.752
Range of post-breakfast glucose elevation (mg/dL)				
Peak	92 ± 86	132 ± 73	50 ± 81	*P* = 0.006**
Postprandial 1-h	67 ± 53	87 ± 47	46 ± 53	*P* = 0.028**
Postprandial 2-h	80 ± 89	113 ± 84	44 ± 83	*P* = 0.028**
Post-breakfast concentration gradient (mg/dL/min)				
Peak	0.97 ± 0.96	1.42 ± 0.72	0.49 ± 0.97	*P* = 0.005**
Postprandial 1-h	1.12 ± 0.89	1.45 ± 0.78	0.77 ± 0.88	*P* = 0.028**
Postprandial 2-h	0.67 ± 0.74	0.94 ± 0.70	0.37 ± 0.69	*P* = 0.028**
Duration of the up-to-peak glucose values after breakfast (min)	89.0 ± 36.3	98.4 ± 33.1	79.0 ± 38.0	*P* = 0.139

Data are expressed as means ± SD.

* Unpaired student's two sampled t-test was employed for comparisons between the hypoglycemic and non-hypoglycemic patients.

** *P*<0.05.

For all patients (n = 31), nocturnal glucose nadir was 87 ± 53 mg/dL. The results of indices for glucose variability before and after breakfast were as follows: I) fasting glucose levels, 112 ± 66 mg/dL; II) post-breakfast peak, 1- and 2-hour glucose levels, 204 ± 60, 179 ± 55 and 192 ± 65 mg/dL; III) range of post-breakfast peak, postprandial 1- and 2-hour glucose elevation, 92 ± 86, 67 ± 53 and 80 ± 89 mg/dL; and IV) post-breakfast peak, postprandial 1- and 2- hour glucose concentration gradient, 0.97 ± 0.96, 1.12 ± 0.89, 0.67 ± 0.74 mg/dl/min (Table 1).

**Fig 2:**
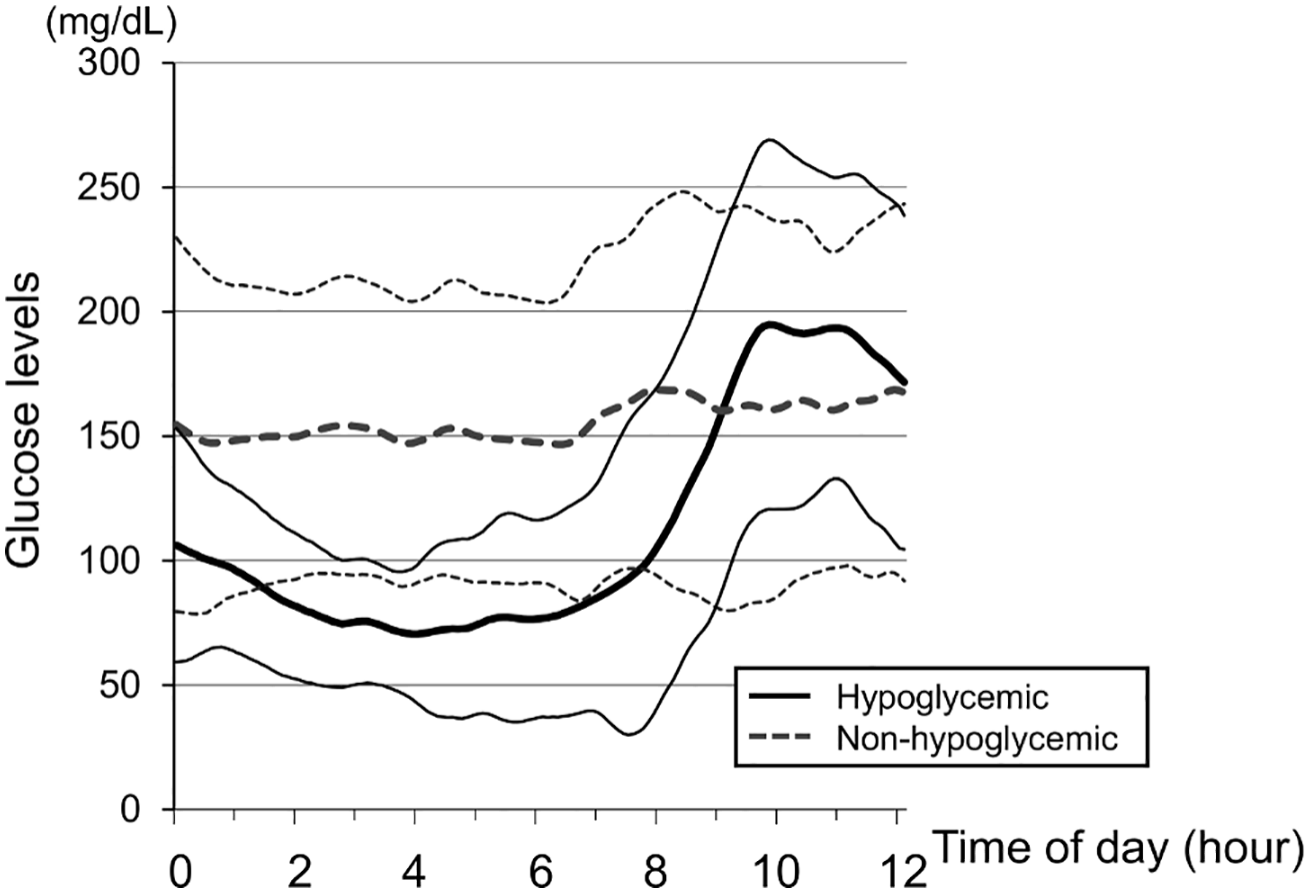
Glucose profiles showing nighttime to post-breakfast glucose levels. Hypoglycemic patients (n = 16); Non-hypoglycemic patients (n = 15). Curves are expressed as means ± standard deviations.

A total of 16 patients (51.6%) reported having nocturnal asymptomatic hypoglycemia (hypoglycemia group) lasting 80 ± 121 minutes, and were not significantly different from those who did not (non-hypoglycemia group) with regard to their age, HbA1c, BMI, total insulin dose used, bolus insulin dose used (as well as bolus insulin dose before breakfast), and basal/bolus insulin ratio. The duration of diabetes was significantly longer in the hypoglycemia group at 23.9 ± 10.3 years compared to 14.9 ± 6.6 years in the non-hypoglycemia group (*P* = 0.008). Hypoglycemia occurred in eleven patients (68.7%) who had received insulin degludec once in the evening or at bedtime and in five patients (31.3%) who had received insulin degludec once in the morning. The frequency of hypoglycemia did not significantly differ depending on the timing of the insulin injection (*P* = 0.16; Fisher’s exact test). Fig 2 shows the glucose profiles from nighttime to post-breakfast in the hypoglycemia and non-hypoglycemia groups.

**Fig 3:**
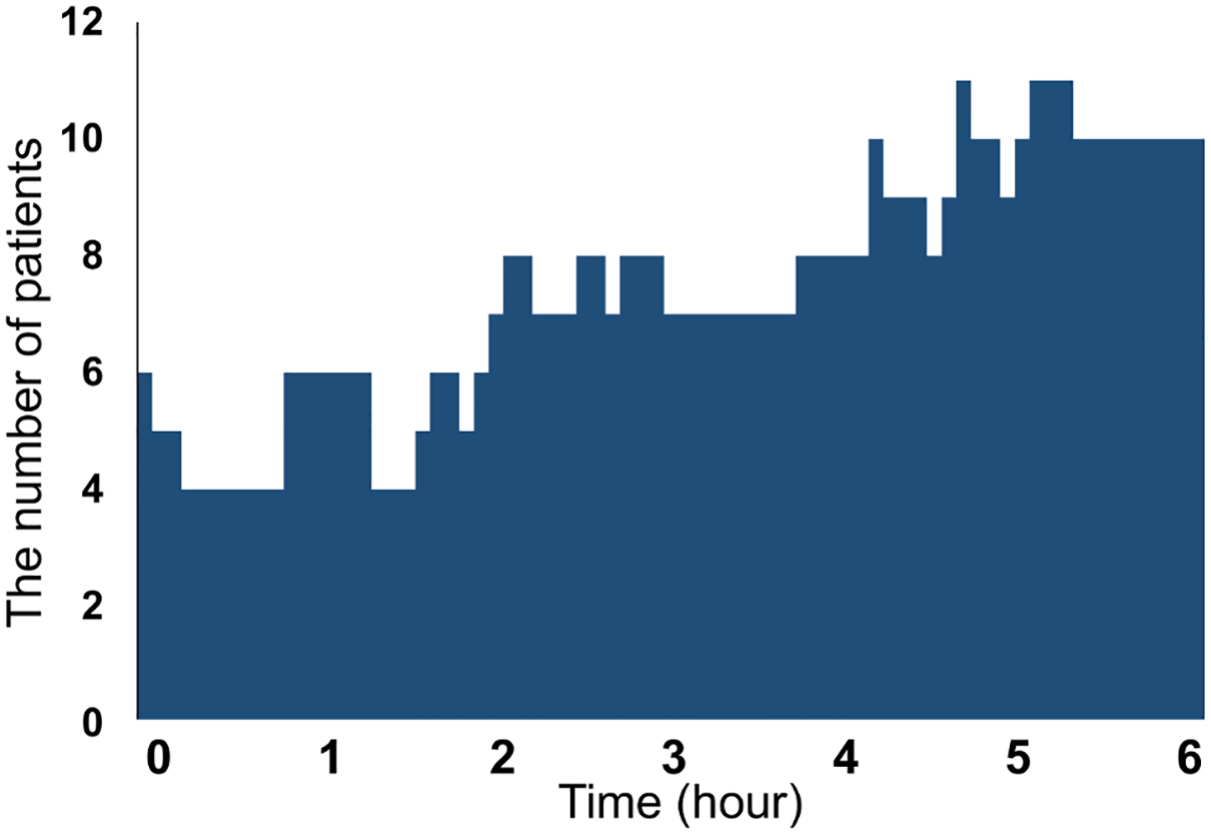
Number of patients experiencing hypoglycemia over time.

In addition, the number of patients experiencing hypoglycemia over time is shown as a bar graph in Fig 3, where the patients are visually represented as having experienced nocturnal hypoglycemia unawareness most often during the hours from 4:00 AM to 6:00 AM.

The indices for glucose variability were compared before and after breakfast between the hypoglycemia and non-hypoglycemia groups (Table 1). Hypoglycemia group had significantly lower fasting glucose levels than non-hypoglycemia group (82 ± 48 mg/dL vs. 144 ± 69 mg/dL; *P* = 0.009). While there was no difference in regard to the post-breakfast peak, 1- and 2-hour glucose levels between the two groups, post-breakfast glucose elevation, defined as the change from pre- to post-breakfast peak, 1- and 2-hour glucose levels, were significantly greater in the hypoglycemia group (peak, 132 ± 73 mg/dL vs. 50 ± 81 mg/dL, *P* = 0.0006; postprandial 1-hour, 87 ± 47 mg/dL vs. 46 ± 53 mg/dL, *P* = 0.028; postprandial 2-hour, 113 ± 84 mg/dL vs. 44 ± 83 mg/dL, *P* = 0.028). The post-breakfast glucose concentration gradients were also significantly greater in the hypoglycemia group than in the non-hypoglycemia group (peak, 1.42 ± 0.72 mg/dL/min vs. 0.49 ± 0.97 mg/dL/min, *P* = 0.005; postprandial1-hour, 1.45 ± 0.78 mg/dL/min vs. 0.77 ± 0.88 mg/dL/min, *P* = 0.028; postprandial 2-hour, 0.94 ± 0.70 mg/dL/min vs. 0.37 ± 0.69 mg/dL/min, *P* = 0.028).

**Fig 4:**
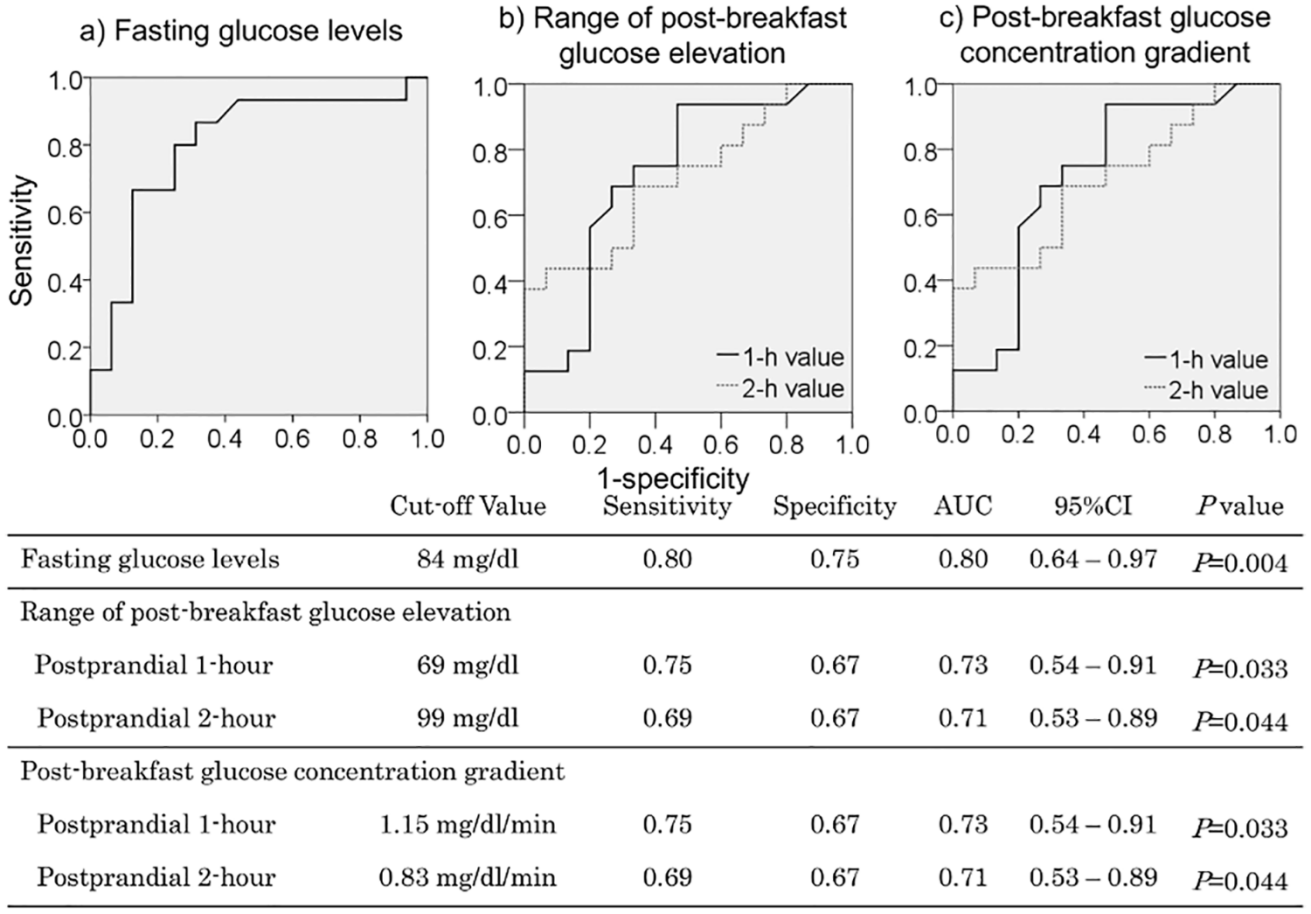
Cut-off values for predicting nocturnal asymptomatic hypoglycemia. 95%CI:95% confidence interval.

The cut-off values obtained by ROC analyses for predicting the occurrence of nocturnal asymptomatic hypoglycemia were fasting glucose levels 84 mg/dL or lower (sensitivity, 0.80; specificity, 0.75; AUC, 0.80; *P* = 0.004); 1-hour post-breakfast glucose elevation 69 mg/dL or higher (sensitivity, 0.75; specificity, 0.67; AUC, 0.73; *P* = 0.033); 2-hour post-breakfast glucose elevation 99 mg/dL or higher (sensitivity, 0.69; specificity, 0.67; AUC, 0.71; *P* = 0.044); the 1-hour post-breakfast glucose concentration gradient 1.15 mg/dL/min or higher (sensitivity, 0.75; specificity, 0.67; AUC, 0.73; *P* = 0.033); and the 2-hour post-breakfast glucose concentration gradient 0.83 mg/dL/min or higher (sensitivity, 0.69; specificity, 0.67; AUC, 0.71; *P* = 0.044) (Fig 4).

Multivariate logistic regression model constructed by the forward selection method (Likelihood Ratio) identified longer duration of diabetes (per 1-year increase in duration of diabetes) (1.19, 1.02–1.40) (hereafter each shown with its adjusted OR and 95% confidence interval) as a sole predictor of nocturnal hypoglycemia unawareness. However, the following were not prediction factors of nocturnal hypoglycemia unawareness: HbA1c (per 1% increase in HbA1c) (1.24, 0.15–10.4); female sex (0.33, 0.03–4.28); BMI (per 1 kg/m^2^ increase in BMI) (1.01, 0.62–1.65); total daily dose of insulin (per 1 U/kg increase in total daily dose of insulin) (1.19, 0.01–193.9); basal insulin ratio (per 1% increase in basal insulin ratio) (1.05, 0.95–1.16); and once daily insulin in the morning (vs. once in the evening or before bed time) (5.71, 0.55–58.9).

## Discussion

Hypoglycemia was noted in 77% of the subjects in the Diabetes Control and Complications Trial (DCCT), with 55% of the hypoglycemic episodes shown to occur during nighttime (2, 9, 31), demonstrating that nocturnal hypoglycemia frequently occurs in patients with type 1 diabetes. In agreement with this finding, nocturnal hypoglycemia unawareness (glucose levels <70 mg/dL) was noted in 16 (51.6%) of the 31 patients in this study whose mean HbA1c was controlled to 7.8 ± 0.7% with frequent injections of insulin degludec.

In this study, no significant difference was noted between the hypoglycemia and non-hypoglycemia groups with regard to their age, HbA1c, BMI, total insulin dose used, bolus insulin dose used (as well as bolus insulin dose before breakfast), basal insulin dose used, and basal/bolus insulin ratio. However, the duration of diabetes was shown to be significantly longer in the hypoglycemia group compared in the non-hypoglycemia group (*P* = 0.008). The DCCT reported that long durations of diabetes can predict the occurrence of serious hypoglycemia [31]. Factors that make these patients susceptible to nocturnal hypoglycemia include decreased autonomic response to hypoglycemia (hypoglycemia-associated autonomic failure) during sleep and decreased insulin-antagonistic hormone response [32–34]. Thus, it was thought to be likely that, in this study, the longer duration of diabetes would account for significantly decreased autonomic response to hypoglycemia associated with the progression of neurological damage, as well as a far greater impaired glucagon response in the hypoglycemia group.

On the other hand, hypoglycemia tended to occur more frequently among those in the hypoglycemia group who were injected with insulin degludec in the evening or before bedtime (eleven patients, 68.7%), compared to those who did so in the morning (five patients, 31.3%). However, the difference was not statistically significant (*P* = 0.16). In this regard, it is of note that insulin degludec is an ultra-long-acting basal insulin that is capable of providing a far more flat pattern of insulin action through its sustained and stable absorption, compared to other long-acting insulin formulations [25]. However, it is also suggested that its pattern of insulin action may vary intra-individually and may not be completely flat [35]. Therefore, the insulin action may have peaked during nighttime, which could have made those who injected insulin degludec in the evening or before bedtime more susceptible to nocturnal asymptomatic hypoglycemia.

Again, the fasting glucose levels were shown to be significantly lower in the hypoglycemia group. Earlier CGM-based studies on patients with type 1 diabetes have reported similar results [22, 23, 36–38]. On the other hand, the glucose elevations from pre- to post-breakfast glucose levels and the post-breakfast glucose gradients were shown to be greater at postprandial 1- and 2-hours in the hypoglycemia group, with the post-breakfast glucose levels greatly and acutely increasing among those with nocturnal hypoglycemia unawareness. This may be accounted for as follows: the use of long-acting insulin degludec led to decreases in fasting glucose levels but was also responsible for nocturnal hypoglycemia unawareness. Therefore, it is likely that it increased counter-regulatory hormones, which led to acute increases in post-breakfast glucose levels in the hypoglycemia group. Similar observations have been reported for insulin detemir and insulin glargine [24].

The ROC analysis-derived cut-offs for estimating the occurrence of nocturnal asymptomatic hypoglycemia included fasting glucose levels < 84 mg/dL. Given that the study included patients with type 1 diabetes with a mean HbA1c value of 7.8 ± 0.7%, which translates into a mean glucose level of 177 mg/dL [39], the cut-off of 84 mg/dL is lower than the HbA1c values. Therefore, it is suggested that those with fasting glucose levels lower than those estimated from their HbA1c values may be suspected of having had hypoglycemia overnight. Again, study results suggest that, even without recourse to CGM assessments, measured pre-breakfast, 1- and 2-hour post-breakfast glucose levels, coupled with measured 1-hour post-breakfast glucose excursions and post-breakfast glucose gradients, may have a close to 70% probability of estimating the occurrence of nocturnal hypoglycemia.

We previously reported that fasting glucose levels below 135 mg/dL and changes from pre-breakfast to 1- and 2-hour post-breakfast glucose levels in excess of 54 mg/dL and 78 mg/dL, respectively, may have a 60% to 80% probability of estimating the occurrence of nocturnal asymptomatic hypoglycemia in inpatients with type 1 diabetes receiving insulin detemir or insulin glargine [24]. The present study differs from the earlier study because it involves outpatients with type 1 diabetes and employed insulin degludec as basal insulin. Interestingly, the cut-off values for the fasting glucose levels for predicting nocturnal asymptomatic hypoglycemia were lower in this study than in the previous study, which may be due to more sustained action of the insulin degludec compared to other insulin formulas. However, it may have important clues to offer in terms of estimating the occurrence of hypoglycemia unawareness in patients with type 1 diabetes receiving insulin therapy, while the meal times, wake-up times, and bedtimes may have differed from one patient to another in this study. Of note, the BEGIN Basal-Bolus Type 1 study [26] found no significant difference between those on insulin degludec and those on insulin glargine (even in terms of HbA1c values) when the fasting glucose levels were controlled to 70 to 89 mg/dL using either type of insulin. Meanwhile, nocturnal hypoglycemia was 25% less frequent among those on insulin degludec. This suggests that, unlike other long-acting insulin formulations, insulin degludec does not increase the risk for nocturnal hypoglycemia even when its dose is adjusted to target fasting glucose levels between 80 and 100 mg/dL. It is interesting to note here that the nocturnal glycemic variability profiles (Fig 2) demonstrated that those without nocturnal hypoglycemia showed a flat pattern with insulin degludec.

The limitations of this outpatient study are that it included a relatively small sample and the breakfast times may have differed from one patient to another. However, pilot studies, such as this one, do provide valuable findings and are required to enable power calculations for future studies, while further study is required in a larger population of patients with type 1 diabetes matched for breakfast time in the future. Again, our proposed cut-off values for these parameters were calculated by using ROC curve analysis and therefore could not completely exclude the false-positive fraction. However, the ROC curve displays the trade-off between sensitivity and specificity and is useful in providing the best combination of diagnostic sensitivity and specificity for predicting nocturnal hypoglycemia unawareness.

The authors hope that the findings reported herein may serve as a benchmark for implementing insulin therapy in patients with type 1 diabetes while simultaneously minimizing the risk for hypoglycemia.

## Supporting information

S1 File Patient and CGM data. (XLSX)
